# Body mass index in the middle-aged offspring of parents with severe mental illness

**DOI:** 10.1017/S0033291722000253

**Published:** 2023-06

**Authors:** Maria Protsenko, Martta Kerkelä, Jouko Miettunen, Juha Auvinen, Marjo-Riitta Järvelin, Peter B. Jones, Mika Gissler, Juha Veijola

**Affiliations:** 1 Department of Psychiatry, Research Unit of Clinical Neuroscience, University of Oulu, Oulu, Finland; 2 Center for Life Course Health Research, University of Oulu, Oulu, Finland; 3 Medical Research Center Oulu, Oulu University Hospital and University of Oulu, Oulu, Finland; 4 Department of Epidemiology and Biostatistics, MRC-PHE Centre for Environment and Health, School of Public Health, Imperial College London, London, UK; 5 Unit of Primary Health Care, Oulu University Hospital, OYS, Oulu, Finland; 6 Department of Psychiatry, University of Cambridge, Cambridge, UK; 7 THL, Department of Knowledge Brokers, Finnish Institute for Health and Welfare, Helsinki, Finland; 8 Research Centre for Child Psychiatry, University of Turku, Turku, Finland; 9 Region Stockholm, Academic Primary Health Care Centre, Stockholm, Sweden; 10 Department of Molecular Medicine and Surgery, Karolinska Institute, Stockholm, Sweden; 11 Department of Psychiatry, University Hospital of Oulu, Oulu, Finland

**Keywords:** Bipolar disorder, body mass index, cohort study, depression, schizophrenia

## Abstract

**Background:**

People with severe mental illness (SMI) have an elevated risk of obesity but the causes and mechanisms are unclear. We explored the familial association between parental SMI and body mass index (BMI) in middle-aged offspring. Our objective was to determine if the offspring of either parent with SMI have an increased risk for obesity.

**Methods:**

The Northern Finland Birth Cohort 1966 is a cohort study of offspring with expected date of birth in 1966. The data include originally 12 068 mothers and 12 231 children from the provinces of Lapland and Oulu in Finland. The final study sample included 5050 middle-aged offspring. Parental SMI was used as exposure in the study. BMI measured at the age of 46 years was used as a primary outcome.

**Results:**

Risk for obesity was elevated in the offspring of mothers with SMI [overweight: adjusted odds ratio (OR) 1.93 (1.29–2.90), obese class I: 1.97 (1.20–3.25), obese classes II–III: 2.98 (1.67–5.33)]. For the offspring of either parent with SMI, statistically significant results were found in obese class I and obese classes II–III [overweight: adjusted OR 1.21 (0.94–1.54), obese class I: 1.52 (1.03–1.08), obese classes II–III: 1.53 (1.01–2.32)].

**Conclusions:**

We found an elevated risk of obesity in the middle-aged offspring of either parent with SMI, especially in the offspring of mothers with SMI. Thus, there might be a common familial pathway leading to the co-occurrence of obesity and SMI.

## Introduction

The prevalence of obesity is an increasing public health problem with significant costs (Ng et al., 2014). People with obesity have an increased risk for a number of common medical conditions, including type 2 diabetes, cardiovascular disease, coronary heart disease, dyslipidaemia and hypertension (McElroy, 2009). The increased physical illness may be associated with unhealthy lifestyles such as smoking, substance use, often with inadequate access to health services (Crawford et al., 2014; Dipasquale et al., 2013; Vancampfort, Probst, Knapen, Carraro, & De Hert, 2012).

People with severe mental illness [SMI; schizophrenia, bipolar disorder, major depressive disorder (MDD)] have a higher prevalence of different physical illnesses. One of the physical conditions that has high prevalence among people with SMI is obesity (De Hert et al., 2011). One factor that induces weight gain is antipsychotic medication (Garriga et al., 2019). Also, people with SMI have been found to be more sedentary and less physically active than controls. However, higher levels of moderate or vigorous physical activity have been reported in Europe in people with SMI (Vancampfort et al., 2017). Some studies suggest that metabolic disturbances such as increased insulin resistance and endothelial dysfunction may exist before the development of SMI and are independent of the use of antipsychotics (Chen et al., 2016). Still, there is lack of knowledge about the transgenerationality of SMI and obesity in families.

Higher somatic morbidity among offspring of parents with SMI has been reported (Davidsen et al., 2021; Ranning et al., 2020). The possible explanatory mechanisms for increased somatic morbidity may be the existence of shared genetic factors for mental disorders and certain somatic diseases, while environmental adversity is also likely to play a role. Parents with SMI face competing demands of attending to the needs of their child and at the same time managing their own mental illness. Previous studies have found that the presence of maternal mental health problems is positively associated with the number of problem-related child healthcare visits. The offspring of parents with a lifetime diagnosis of SMI have also an increased risk of missing well-child healthcare visits and vaccinations compared to the offspring of parents without SMI (Davidsen et al., 2021). This may affect offspring physical health in their late adulthood.

MDD and obesity in adolescence have been previously studied, and they are known to be associated (Marmorstein & Iacono, 2016). Having one of these conditions increases the risk of having the other condition during lifetime (Atlantis & Baker, 2008; Faith et al., 2011). Obesity and MDD have both genetic and environmental risk factors but very little is known about the possible association between MDD in parents and obesity in their offspring (Stice, Presnell, Shaw, & Rohde, 2005; Sullivan, Neale, & Kendler, 2000). There are several reasons why obesity and MDD may co-exist within families. MDD in parents may create an environmental context that predisposes offspring to both conditions. Parents may have poor self-esteem and unhealthy eating habits which could contribute to comorbidity (Marmorstein & Iacono, 2016). In addition, environmental factors that affect the family such as poverty may contribute to the risk for these conditions among family members. Also shared biological factors such as dysfunction of stress response system (hypothalamic-pituitary-adrenal axis) or chronic inflammation may contribute to comorbidity in families (Bornstein, Schuppenies, Wong, & Licinio, 2006; Shelton & Miller, 2010). Obesity and SMI are both associated with dysfunction of stress response system and chronic inflammation (Firth et al., 2019).

The objective of this study was to study the risk for obesity in the offspring of parents with SMI. We explored if the risk for obesity is increased among the offspring of either parent with SMI compared to comparison group in the Northern Finland Birth Cohort 1966 (NFBC1966 and NFBC1986, 2021). Our hypothesis was that the offspring of people with SMI have an increased risk for obesity.

## Materials and methods

### Study design

The Northern Finland Birth Cohort 1966 (NFBC1966 and NFBC1986, 2021) is a prospective follow-up study of offspring with expected date of birth in 1966. The data include originally 12 068 mothers and 12 231 children from the provinces of Lapland and Oulu in Finland (NFBC1966 and NFBC1986, 2021). We used register data from 1969 until offspring's age of 46 years. We also used personal identification codes given to all citizens and residents of Finland to merge register data to the NFBC1966 data set. Permission to link register data was obtained from the Ministry of Social Affairs and Health. The ethical committee of the Northern Ostrobothnia Hospital District keeps the study under review.

### Participants

**Fig. 1.**
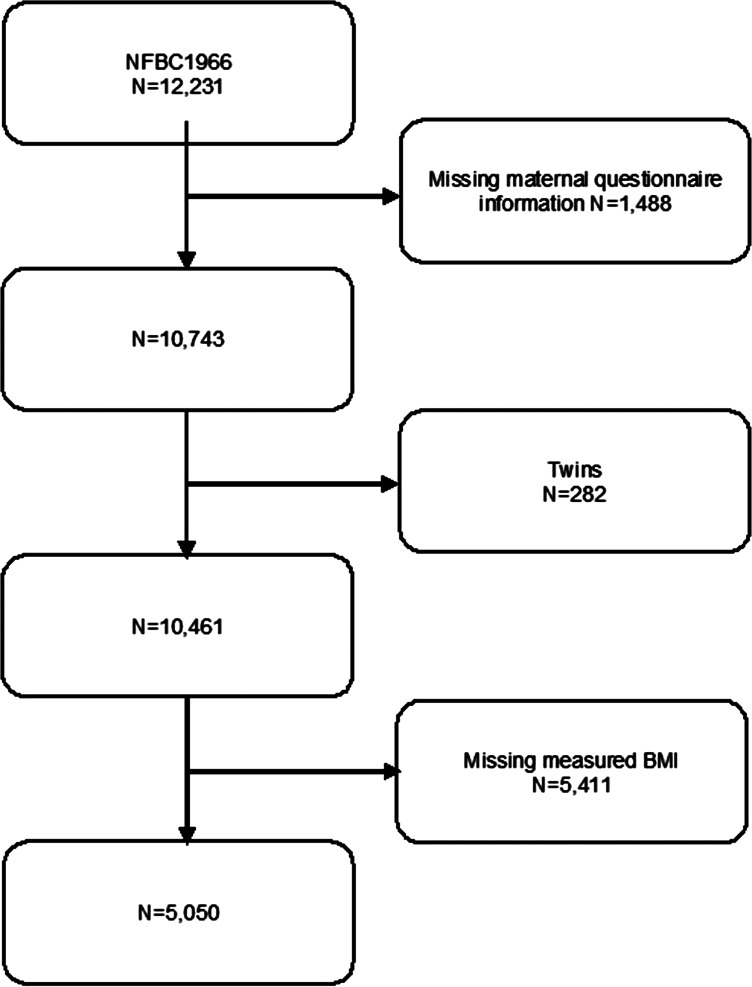
Flowchart of study design.

We excluded 1488 (12.2%) members of the cohort whose maternal questionnaire information was missing for any variable used in the study. We also excluded 282 (2.3%) twins and 5411 (44.2%) whose weight or height was not measured in clinical examination at the age of 46 years. Our final study sample included 5050 offspring (Fig. 1). This sample included 388 (7.7%) offspring who had a parent with SMI and 4662 (92.3%) offspring in comparison cohort. All participants gave written informed consent.

### Classification for obesity

Weight (cm) and height (cm) were measured in a clinical examination and body mass index (BMI) was calculated at the offspring age of 46 years. The BMI is a simple index of weight-for-height that is commonly used to classify underweight, overweight and obesity in adults. It is calculated as weight in kilograms divided by the square of the height in meters (kg/m^2^). The international classification of adult underweight, overweight and obesity according to BMI was used, as given in Table 1 (WHO: Global Database on Body Mass Index, 2006).

**Table 1.** The international classification of adult underweight, overweight and obesity based on BMI

Classification	BMI (kg/m^2^)
Underweight	<18.5
Normal	18.5–24.9
Overweight	25.0–29.9
Obese class I	30.0–34.9
Obese classes II–III	⩾35

### Parental SMI

We used parental SMI as exposure in the study. The information of parental SMI was obtained from the Care Register for Health Care (CRHC), kept by THL, the Finnish Institute for Health and Welfare. Parents with SMI were those who had been diagnosed for any hospital treated psychiatric disorder during 1969–1982 (at the offspring age of 3–16 years, ICD-8 codes 290–315). Maternal and paternal SMI were studied separately. The CRHC has a very good accuracy and it is one of the oldest person-level hospital discharge registers (Sund, 2012).

### Confounders

Information about the confounders was obtained from the mother by the local midwives using a pre-defined questionnaire in the antenatal clinics. The questionnaire was filled in from the 24th to 28th gestational week. If this was impossible, the questionnaire was completed later during the pregnancy or after the delivery.

We used offspring gender, maternal smoking, mother's marital status, mother's educational level and maternal BMI as confounders. Maternal smoking was dichotomized into smokers and non-smokers. Mothers' marital status was dichotomized into two groups: married and others (unmarried, divorced, widowed). Those mothers who continued smoking in the second trimester were considered as smokers. Mothers' educational level was categorized into three levels according to the length of educations: less than 9, 9–11, and 12 years or more. Mother's weight (kg) and height (cm) before pregnancy were asked in a pre-defined questionnaire at antenatal clinic. BMI was calculated using these measures.

### Statistical methods

Analyses were performed using R version 1.2.1335. Descriptive statistics were used to compare the offspring of people with SMI and comparison group. Comparisons were conducted using cross-tabulation and χ^2^ testing. We were interested in the five BMI categories. We calculated crude odds ratios (OR) and adjusted OR using multinomial logistic regression. We excluded ‘Underweight’ class from our regression analyses, while this event was too rare. We used gender, marital status, mother's education, mother's smoking during pregnancy and mother's BMI as confounding factors. Mothers' educational level was categorized into two levels (less than 9 years and over 9 years) in regression analyses as the event was too rare for highest education level. We calculated the crude OR and adjusted OR with 95% confidence intervals (CI) for each exposed group (offspring of mothers with SMI, offspring of fathers with SMI and offspring of either parent or both with SMI).

## Results

### Descriptive analyses

Table 2 summarizes the characteristics of middle-age offspring and their mothers in the NFBC1966 divided into two groups: offspring of either parent with SMI and comparison group. Mothers of the offspring of parents with SMI were more likely to continue smoking during pregnancy.

**Table 2.** Characteristics of offspring and their mothers – separately in the offspring of either parent with severe mental illness (SMI), offspring of mothers with SMI, offspring of fathers with SMI and comparison group (offspring of parents without SMI)

	Offspring of either parent with SMI *N* (%)			Offspring of mothers with SMI *N* (%)			Offspring of fathers with SMI *N* (%)		
	Yes 388	No 4662	χ^2^ (*p* value)	Yes 156	No 4894	χ^2^ (*p* value)	Yes 246	No 4804	χ^2^ (*p* value)
*Offspring's characteristics*									
Sex									
Male	160 (41.2)	2061 (44.2)	1.17 (0.280)	59 (37.8)	2162 (44.2)	2.23 (0.136)	105 (42.7)	2116 (44.0)	0.13 (0.723)
Female	228 (58.8)	2601 (55.8)		97 (62.2)	2732 (55.8)		141 (57.3)	2688 (56.0)	
*Maternal characteristics*									
Smoked during pregnancy	65 (16.8)	583 (12.5)	5.40 (0.020)	30 (19.2)	618 (12.6)	5.32 (0.021)	39 (15.9)	609 (12.7)	1.84 (0.175)
Marital status									
Married	373 (96.1)	4526 (97.1)	0.81 (0.369)	145 (92.9)	4754 (97.1)	7.77 (0.005)	242 (98.4)	4657 (96.9)	1.20 (0.273)
Other	15 (3.9)	136 (2.9)		11 (7.1)	140 (2.9)		4 (1.6)	147 (3.1)	
Education level									
<9 years	258 (66.5)	2876 (61.7)	3.62 (0.164)	102 (65.4)	3032 (62.0)	2.02 (0.364)	163 (66.3)	2971 (61.8)	1.98 (0.372)
9–11 years	112 (28.9)	1519 (32.6)		49 (31.4)	1582 (32.3)		70 (28.5)	1561 (32.5)	
⩾12 years	18 (4.6)	267 (5.7)		5 (3.2)	280 (5.7)		13 (5.3)	272 (5.7)	
BMI [mean (s.d.)]	23.02 (2.86)	23.16 (3.22)	−0.93 (0.401)^a^	23.20 (2.92)	23.15 (3.20)	0.22 (0.843)^a^	22.85 (2.82)	23.17 (3.21)	−1.70 (0.130)^a^

a *t* test (*p* value).

Table 3 summarizes the distribution in BMI between the two groups: offspring of either parent with SMI and without SMI. There was no statistically significant difference in BMI categories between the two groups (χ^2^ = 9.45, *p* = 0.051).

**Table 3.** Body mass index in the offspring of either parent with severe mental illness (SMI) and without SMI

	Offspring of either parent with SMI *N* = 388	Comparison group *N* = 4662	χ^2^ (*p* value)
	*N* (%)	*N* (%)	
Underweight	4 (1.0)	30 (0.6)	(χ^2^ = 9.45, *p* = 0.051)
Normal range	128 (33.0)	1814 (38.9)	
Overweight	152 (39.2)	1833 (39.3)	
Obese class I	73 (18.8)	699 (15.0)	
Obese classes II–III	31 (8.0)	286 (6.1)	

### Multinomial logistic regression analyses

In Table 4 is presented the risk of obesity in three different categories (overweight, obesity class I, obesity classes II–III) for the offspring of mothers with SMI, for the offspring of fathers with SMI and for the offspring of either parent with SMI. Analyses could not be conducted separately for male and female offspring as the exposure was too rare. Statistically significant results were found for the offspring of mothers with SMI [overweight: adjusted OR 1.93 (95% CI 1.29–2.90), obese class I: adjusted OR 1.97 (1.20–3.25), obese classes II–III: adjusted OR 2.98 (1.67–5.33)]. For the offspring of fathers with SMI, the results were not statistically significant [overweight: adjusted OR 0.97 (0.72–1.32), obese class I: 1.41 (0.98–2.03), obese classes II–III: 0.87 (0.48–1.58)]. For the offspring of either parent with SMI, statistically significant results were found in obese class I and obese classes II–III [overweight: adjusted OR 1.21 (0.94–1.54), obese class I: 1.52 (1.03–1.08), obese classes II–III: 1.53 (1.01–2.32)].

**Table 4.** Crude odds ratios (OR) and adjusted OR with 95% confidence interval estimates predicting the risk of obesity

	Body mass index		
	Overweight	Obese class I	Obese classes II–III
Offspring of mothers with SMI			
*N*	70	29	18
Crude OR^b^	1.83 (1.23–2.73)	1.96 (1.20–3.19)	3.02 (1.70–5.35)
Adjusted OR^a^^,^^b^	1.93 (1.29–2.90)	1.97 (1.20–3.25)	2.98 (1.67–5.33)
Offspring of fathers with SMI			
*N*	90	48	13
Crude OR^b^	0.95 (0.71–1.29)	1.33 (0.93–1.91)	0.86 (0.48–1.56)
Adjusted OR^a^^,^^b^	0.97 (0.72–1.32)	1.41 (0.98–2.03)	0.87 (0.48–1.58)
Offspring of either parent with SMI			
*N*	152	73	31
Crude OR^b^	1.18 (0.92–1.50)	1.48 (1.10–2.00)	1.53 (1.02–2.31)
Adjusted OR^a^^,^^b^	1.21 (0.94–1.54)	1.52 (1.03–1.08)	1.53 (1.01–2.32)

a Adjusted for gender, mothers' smoking during pregnancy, mothers' marital status, mothers' education (two classes) and mothers BMI.

b Normal range BMI is used as reference group.

## Discussion

### Main findings

We found an elevated risk of substantially increased BMI in the middle-aged offspring of either parent with SMI, especially in the offspring of mothers with SMI. However, we found no elevated risk of increased BMI in the middle-aged offspring of fathers with SMI. This means that there might be a common familial pathway leading to the co-occurrence of obesity and SMI.

### Comparison to earlier studies

According to our knowledge, this is the first study to focus on BMI in the middle-aged offspring of parents with SMI. Socioeconomic status, parental education and family structure have been shown to influence offspring BMI (Chen & Escarce, 2010; Lamerz et al., 2005; Shrewsbury & Wardle, 2008). Maternal smoking during pregnancy may also be associated with offspring future obesity (Ino, 2010). That is why mother's marital status and mothers' educational level and maternal smoking were used as confounders.

Higher somatic morbidity among the offspring of parents with SMI has been reported (Davidsen et al., 2021; Ranning et al., 2020). Davidsen et al. found that parents with SMI are less compliant with preventive child healthcare activities than parents without SMI and are this way exposed to increased somatic morbidity. However, they did not study specifically obesity and they had follow-up time only until the offspring age of 6 years. Ranning et al. investigated the full spectrum of somatic illness in offspring and found increased incidences for most disease categories (Ranning et al., 2020). They did not focus on obesity but had quite long follow-up time until the offspring age of 30 years. Baptista et al. observed that relatives of bipolar patients tended to be more prone to obesity than the relatives of schizophrenic patients (Baptista et al., 2011). However, they focused on first-degree relatives. These study results are in line with our findings suggesting an increased risk for obesity in the offspring of parents with SMI. Compared to these previous studies, we had extensive follow-up time until the offspring age of 46 years.

One study examined if BMI is higher among unaffected first-degree-siblings of patients with SMI and found no significant difference between siblings and controls (Chouinard et al., 2018). Spelman et al. observed that patients with schizophrenia had a lower BMI than their first-degree relatives or control subjects. Also, none of the three groups were obese. However, there was no difference in BMI between first-degree relatives and control subjects (Spelman, Walsh, Sharifi, Collins, & Thakore, 2007). These results by Chouinard et al. and Spelman et al. are in not in line with our findings. However, both of the studies did not study specifically offspring and both had rather small sample size.

The possible explanatory mechanisms for increased obesity may be the existence of shared genetic factors for mental disorders and certain diseases, while environmental adversity is also likely to play a role (Ranning et al., 2020). Parents have an important role in the development of child's healthy lifestyles and in that way affect child's weight (Golan & Crow, 2004). A range of family practices during infancy, childhood and adolescence affecting child's weight development have been studied, including breastfeeding, early introduction of solid foods, parental feeding practices and physical activity parenting (Edwardson & Gorely, 2010; Hoyos Cillero & Jago, 2010; Loprinzi, Cardinal, Loprinzi, & Lee, 2012; Rodgers et al., 2013). While these studies have focused on infancy, childhood and adolescence, little is known about offspring's weight in middle-aged offspring. It has also been suggested that SMI might in some cases be linked to a dysfunction in the immune system, which may also affect the child's risk of general medical illnesses (Benros et al., 2011, 2013; Lydholm et al., 2019). However, in our current study, it was not possible to distinguish clearly the contribution of environmental or genetic factors.

### Strengths

The present study has several strengths. First, we had the possibility to study the long-term effect of parental SMI to offspring BMI until the offspring age of 46 years. Second, the parental SMI was based on data from nationwide register CRHC which has high quality (Sund, 2012). Third, the data of height and weight were measured in clinical examination which increases validity of the BMI measures compared to self-reported data. We also had the possibility to use multiple confounders including maternal BMI, maternal smoking during pregnancy and socioeconomic factors. Fourth, the sample size was relatively large. The risk group and the comparison group were born in the same area in Finland and in the same year.

### Limitations

The present study has few limitations. First, some of the collected maternal information was collected via questionnaire. This may have caused information bias to the study results. Second, we were not able to study parental SMI before pregnancy, during pregnancy or in the infancy of the offspring from 1966 until 1968 due to lacking data. The CRHC did not have complete registration of personal identification codes before 1969. This means that there is a 2–3-year gap on parental diagnoses. Third, socioeconomic factors, such as marital status and smoking, can be on causal pathway in that parental SMI may negatively affect mother's educational level. Fourth, we had no information on childhood BMI so it was not possible to study more specifically developmental period.

## Conclusions

According to our knowledge, this is the first study of BMI in middle-aged offspring of parents with SMI. The findings supported our expectations. We found an elevated risk of substantially increased BMI in the middle-aged offspring of mothers and either parent with SMI. However, we did not find an elevated risk of increased BMI in the middle-aged offspring of fathers with SMI. The findings support our hypothesis of a higher risk for obesity in the offspring of parents with SMI.
